# Pembrolizumab-Induced Ketoacidosis: A Case Report

**DOI:** 10.7759/cureus.65906

**Published:** 2024-07-31

**Authors:** Francisco Guimarães, Nataliya Polishchuk, Cláudia Almeida Martins

**Affiliations:** 1 Internal Medicine, Hospital CUF Descobertas, Lisbon, PRT; 2 Family and Community Medicine, USF AlbaSaúde, Sintra, PRT

**Keywords:** diabetic ketoacidosis, immune checkpoint inhibitors, immunotherapy, diabetes, pd-1 inhibitor, immune-related adverse event

## Abstract

Immune checkpoint inhibitors (ICIs), such as pembrolizumab, have transformed immuno-oncology by demonstrating efficacy against various cancers, including small-cell lung carcinoma and HER2-positive gastric cancer. Despite their benefits, ICIs can provoke immune-related adverse events, with endocrinopathies being rare but carrying significant complications. We report a case of pembrolizumab-induced diabetic ketoacidosis (DKA) in an 87-year-old woman with advanced gastric carcinoma and no prior diabetes history. The patient presented with acute hyperglycemia and metabolic acidosis and was found to have low C-peptide levels without other autoimmune markers typically associated with type 1 diabetes. This case highlights the need for awareness of pembrolizumab as a potential trigger for DKA, especially in patients without a prior diabetes diagnosis. While the management of DKA remains the same, identifying the precipitating factor allows for a comprehensive diagnostic workup and effective long-term management, maintaining the patient's quality of life. This case highlights the complexities of managing DKA in ICI therapy and illustrates the importance of distinguishing between classical DKA presentations and those related to ICIs.

## Introduction

Immune checkpoint inhibitors (ICIs) are targeted, cell-directed therapies first approved in 2011 [1]. They have heralded a new era in immuno-oncology, demonstrating benefits in various cancers, including small-cell lung carcinoma and HER2-positive (HER2+) gastric cancer [1,2]. Pembrolizumab, an immunoglobulin G4-kappa programmed death-1 (PD-1) inhibitor, has been approved in the European Union for treating multiple cancer types such as melanoma, non-small cell lung cancer, classical Hodgkin lymphoma, urothelial cancer, head and neck cancer, renal cell carcinoma, esophageal cancer, gastric cancer, breast cancer, endometrial carcinoma, and cervical cancer. Pembrolizumab prevents the expression of the programmed death-ligand 1 (PD-L1) found on tumor cells, facilitating their identification and destruction through T-cell activation. This mechanism has proven effective, with studies reporting response rates of approximately 22% for advanced gastric cancers [3]. The rate of immune-related adverse events (IRAEs) is relatively low, estimated from 0.3% to 1.3% according to recent systematic reviews [4], primarily involving pneumonitis, hepatitis, and neurotoxic events. However, considering the relatively recent introduction of this drug, the long-term side effects require further study, especially as usage increases. We present a rare case of pembrolizumab-induced diabetic ketoacidosis (DKA) in a patient with advanced gastric carcinoma without a prior diabetes diagnosis.

## Case presentation

An 87-year-old woman presented to the emergency department with acute onset of tiredness, anorexia, asthenia, and hyperglycemia. She reported experiencing polydipsia and polyuria but denied nausea, vomiting, chest pain, abdominal pain, diarrhea, fever, or chills. Her medical history included HER2+ gastric carcinoma diagnosed in 2015, for which she had undergone six cycles of chemotherapy. In 2021, she was diagnosed with metastatic liver lesions. Prior to symptom onset, she had received one cycle of pembrolizumab. She reported no prior diagnosis of diabetes, and her home medications included omeprazole 20 mg, atorvastatin 10 mg, and pembrolizumab. She reported no glucocorticoid use. Her social history revealed no alcohol, tobacco, or drug abuse, and there was no family history of endocrinopathies.

**Figure 1:**
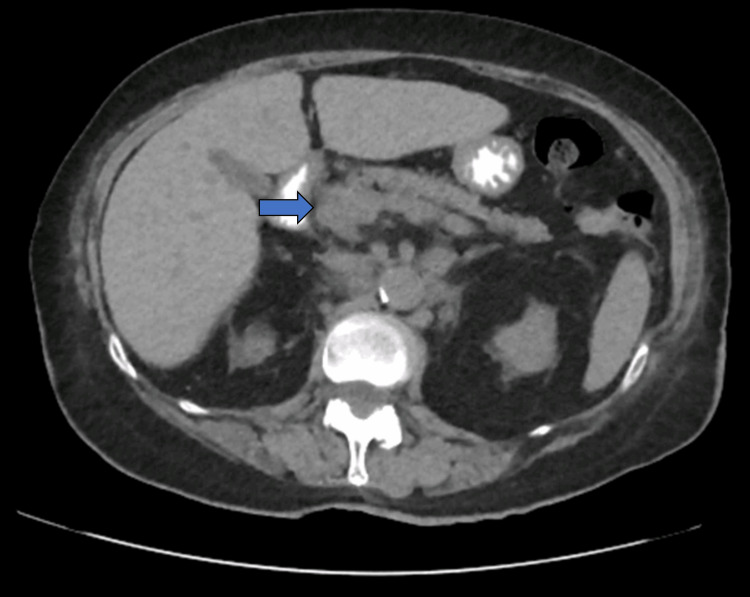
Abdominal CT scan showing the pancreas moderately atrophic with homogeneous parenchyma (blue arrow indicates the cephalic portion) Abbreviation: CT, computed tomography.

Physical examination findings were notable for a fatigued and dehydrated appearance, body mass index of 22 kg/m2, polypnea, blood pressure of 152/78 mmHg, and oxygen saturation of 97%. Laboratory tests indicated a blood glucose level marked as 'HI,' ketonemia marked as 'HI,' and benign results from abdominal, cardiac, and pulmonary examinations. Initial laboratory values showed elevated blood glucose, hyponatremia, creatinine, and arterial blood gas, indicative of metabolic acidosis with an elevated anion gap and low bicarbonate, consistent with DKA. Urinalysis confirmed high glucose and ketones. An abdominal computed tomography (CT) scan described a moderately atrophic pancreas with homogeneous parenchyma (Figure 1). The patient was admitted to the intensive care unit (ICU) for DKA management.

Treatment included volume resuscitation and initiation of an insulin infusion. After three days in the ICU, she was transferred to a medical ward following a transition to subcutaneous insulin. Notably, her C-peptide levels were low, and tests for thyroid-stimulating hormone, free thyroxine, adrenocorticotropic hormone, cortisol, anti-glutamic acid decarboxylase (anti-GAD), anti-tyrosine phosphatase, anti-insulin, and anti-Langerhans islet α cells were all negative (Table 1). After receiving education on insulin and caloric management, she was discharged and referred to internal medicine for diabetes management and a nutritionist for dietary advice. She was prescribed long-acting insulin (glargine) at 26 units. Following a consultation with her oncologist, pembrolizumab was discontinued, but insulin therapy continued.

**Table 1:** Relevant laboratory results Abbreviations: HbA1c, glycated hemoglobin; TSH, thyroid stimulating hormone; FT4, free thyroxine; FT3, free triiodothyronine; GAD, glutamic acid decarboxylase; NA, not applicable.

Laboratory Values	Patient Results	Reference range
Glucose (mg/dl)	575	70-110
Sodium (mmol/L)	129.8	135-145
Chloride (mmol/L)	97	96-108
Potassium (mmol/L)	4.2	3.5-5.1
Bicarbonate (mmol/L)	12.5	20-26
pCO2 (mmHg)	24.5	32-45
pH	7.326	7.350-7.450
HbA1c (%)	9.6	4.3-6.1
Creatinine (mg/dl)	1.41	0.55-1.02
TSH (mUI/L)	2.44	0.35-5.5
FT4 (ng/dL)	1.47	0.8-1.76
FT3 (pg/mL)	2.37	2.3-4.2
C-peptide (ng/mL)	0.5	1.1-4.4
Anti-tyrosine phosphatase	Negative	NA
Anti-insulin	Negative	NA
Anti-GAD	Negative	NA
Anti-Langerhans islet α cells	Negative	NA

## Discussion

Adverse effects of medications are a primary reason for discontinuation, especially with newer drugs where many side effects remain undescribed or statistically insignificant to warrant rejection. IRAEs associated with PD-1 inhibitors have increased since their global approval. Recent publications have begun to elucidate their mechanisms [5,6]. Systematic reviews and meta-analyses have identified fatigue, diarrhea, and rash as common side effects [6], although these occur less frequently than traditional chemotherapy. Specifically, pembrolizumab has shown a 60% incidence of side effects, with severe end-organ toxicities such as myocarditis, pneumonitis, and hepatitis comprising only 0.36% of cases [5,6]. Among these, endocrinopathies represent approximately 2% [5,6]. DKA was reported in just 0.1% of cases [7].

We report a rare case of pembrolizumab-induced DKA in an elderly patient with no previous diabetes diagnosis. The autoimmune workup revealed impaired endogenous insulin production, indicated by low C-peptide levels, a condition typical in type 1 diabetes (T1D) and insulin-dependent type 2 diabetes, and associated with poorer glycemic control outcomes [8]. The pathophysiology of IRAEs remains elusive, but recent studies suggest mechanisms such as antibody-dependent cell-mediated cytotoxicity and complement pathway activation. Most documented cases of pembrolizumab-associated DKA featured anti-GAD positive antibodies (50%), with a lower prevalence of other antibodies (islet antigen 2 [15%], anti-insulin [16%]). Glycated hemoglobin levels are typically low at onset, around 7.3%, which does not correlate with glycemic values at presentation [8,9].

It is necessary to differentiate between T1D and ICI-related diabetes, as they are distinct entities despite their similarities. Fulminant T1D usually presents with digestive or flu-like symptoms and elevated serum pancreatic enzyme levels [9], unlike ICI-related diabetes. Recognizing ICIs as a potential cause of DKA, particularly in patients undergoing immunotherapy for cancer, is essential. The onset of IRAEs, especially endocrinopathies, can vary from weeks to months after the initial medication dose and may persist long after its discontinuation. While DKA management remains largely consistent, outpatient management, which includes long-acting insulin, dietary control, and autoimmune evaluation, is vital to determine the cause.

## Conclusions

ICIs represent a novel and increasingly popular form of immunotherapy due to their superior efficacy and fewer side effects. Their expanding use has led to more frequent reports of IRAEs. Current data indicate that endocrinopathies are a rare but significant side effect of ICIs, increasingly recognized as precipitators of DKA through mechanisms still under investigation. This case underscores the similarities and differences in DKA presentations concerning ICIs versus classical T1D. Typically, DKA in T1D involves serum anti-GAD, insulin autoantibodies, and anti-Langerhans islet α cells. The absence of these antibodies should prompt consideration of an IRAE. Additionally, the patient's atypical presentation, lacking elevated serum pancreatic enzymes and positive antibodies associated with T1D, enhances our understanding of the complex interactions between ICIs and immune regulation.
